# Structural Lessons From the Mutant Proinsulin Syndrome

**DOI:** 10.3389/fendo.2021.754693

**Published:** 2021-09-30

**Authors:** Balamurugan Dhayalan, Deepak Chatterjee, Yen-Shan Chen, Michael A. Weiss

**Affiliations:** Department of Biochemistry and Molecular Biology, Indiana University School of Medicine, Indianapolis, IN, United States

**Keywords:** protein folding, protein structure, folding efficiency, hormone, metabolism

## Abstract

Insight into folding mechanisms of proinsulin has been provided by analysis of dominant diabetes-associated mutations in the human insulin gene (*INS*). Such mutations cause pancreatic β-cell dysfunction due to toxic misfolding of a mutant proinsulin and impairment in *trans* of wild-type insulin secretion. Anticipated by the “Akita” mouse (a classical model of monogenic diabetes mellitus; DM), this syndrome illustrates the paradigm endoreticulum (ER) stress leading to intracellular proteotoxicity. Diverse clinical mutations directly or indirectly perturb native disulfide pairing leading to protein misfolding and aberrant aggregation. Although most introduce or remove a cysteine (Cys; leading in either case to an unpaired thiol group), non-Cys-related mutations identify key determinants of folding efficiency. Studies of such mutations suggest that the hormone’s evolution has been constrained not only by structure-function relationships, but also by the susceptibility of its single-chain precursor to impaired foldability. An intriguing hypothesis posits that *INS* overexpression in response to peripheral insulin resistance likewise leads to chronic ER stress and β-cell dysfunction in the natural history of non-syndromic Type 2 DM. Cryptic contributions of conserved residues to folding efficiency, as uncovered by rare genetic variants, define molecular links between biophysical principles and the emerging paradigm of *Darwinian medicine*: Biosynthesis of proinsulin at the edge of non-foldability provides a key determinant of “diabesity” as a pandemic disease of civilization.

## Introduction 

The Centennial of insulin’s discovery (1) coincides with renewed interest in cellular mechanisms of biosynthesis. The mature hormone is the post-translational product of a single-chain precursor, proinsulin (2, 3). Diverse dominant mutations in the human insulin gene (*INS*) have been identified associated with diabetes mellitus (DM) (4–10). Such mutations impair oxidative folding of nascent proinsulin in the endoplasmic reticulum (ER) of pancreatic β-cells (11, 12). Originally identified as a monogenic cause of permanent neonatal-onset DM (7, 13–15), this syndrome (designated mutant *INS*-gene-induced diabetes of youth; MIDY) can also present in childhood (16) or adolescence (17) (maturity-onset diabetes of the young; MODY). Such variation in onset is ascribed to mutation-specific differences in extent of perturbed folding (12, 18). The spectrum of phenotypes may also reflect polygenic differences in how the β-cell responds to chronic ER stress (19, 20).

MIDY patients are heterozygous. Although one wild-type (WT) insulin allele would ordinarily be sufficient to maintain metabolic homeostasis, studies of the Akita mouse [a corresponding mouse model (21–23)] first demonstrated biochemical dominance: misfolding of the variant proinsulin impairs wild-type (WT) biosynthesis (24, 25). Analogous biochemical interference occurs in β-cell lines (11, 12, 18). ER stress leads to distorted organelle architecture, impaired glucose-stimulated β-cell secretion and eventual cell death (26, 27). Discovery of the mutant proinsulin syndrome has stimulated renewed interest in structural mechanisms of disulfide pairing (28–33) as a critical step in the biosynthesis of insulin (2, 3, 34). The central importance of such mechanisms—both in β-cells and as a general model for oxidative protein folding—have motivated extensive cell-based and animal studies (35–39). Together, these efforts have deepened the biophysical understanding of classical structure-functional relationships in the insulin molecule (9, 10, 19, 40) in relation to cellular mechanisms of biosynthesis (4, 10, 41, 42).

**Figure 1:**
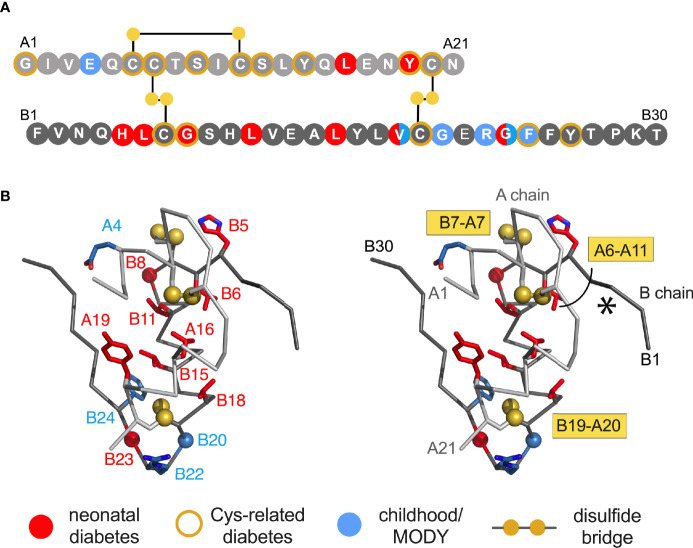
Clinical mutations in *INS* gene. **(A)** Sequence of insulin showing positions of clinical mutations. Residues are labelled by standard single letter code (bold white). The A chain is shown as light gray circles (upper sequence), and B chain as dark gray circles (lower sequence). Color code: neonatal- or delayed onset is indicated by filled red or blue circles, respectively. Sites of Cys-related mutations are highlighted by gold borders (**Tables 1A, B**). Disulfide bridges are indicated by filled gold circles connected by black lines. **(B)** Stereo view of insulin monomer (C_α_-trace ribbon model; PDB entry 4INS) (47). Non-Cys-related mutations are highlighted as in **(A)**; side chains are shown in red as labeled (**Tables 1C, D**). The C_α_ atoms of Gly^B8^, Gly^B20^ and Gly^B23^ are respectively shown as red, blue and red spheres (one-third Van der Waals radii), and sulfur atoms likewise as gold spheres. The A- and B chain ribbons are shown in light and dark gray, respectively. For clarity, symbols are also defined at bottom.

The goal of this review is to provide a structural perspective on *INS* mutations in human proinsulin [for clinical background and history of discovery, see (43)]. A starting point is provided by a general biophysical paradigm: that key interactions in intermediate stages of protein folding often foreshadow spatial relationships in the native state (44–46). Accordingly and in the reverse direction, we will regard the classical crystal structure of insulin (47) as a framework for interpreting folding mechanisms. Given this context, we will restrict our attention to mutations in (or adjoining) the well-organized insulin moiety of proinsulin (48) (**Figure 1A** and **Table 1**). Whereas traditional structure-activity relationships (SAR) pertain to receptor binding (9), contributions of the same residues to folding efficiency may be inapparent once the native structure is reached. The growing MIDY/MODY database of *INS* mutations (**Figure 1B** and **Table S1**) may be exploited to decipher this hidden layer of meaning. As a seeming paradox in Darwinian medicine (49, 50), the biophysical non-robustness of proinsulin biosynthesis suggests that the hormone has evolved to the precarious edge of foldability (40, 51, 52). We envisage that foundational principles of protein folding, structure and stability will be found to rationalize the distribution of MIDY/MODY mutations and broad spectrum of clinical presentations.

**Table 1:** Sites of clinical mutations in proinsulin^a,b^.

**A) removal of a Cys** Cys31 [B7]	Tyr
Cys43 [B19]	Gly, Ser, Tyr, Ala
Cys95 [A6]	Tyr, Ser
Cys96 [A7]	Arg, Ser, Tyr
Cys100 [A11]	Tyr
Cys109 [A20]	Tyr, Phe, Arg
***B) addition of a Cys*** ^c^	
Gly32 [B8]	Cys
Phe48 [B24]	Cys
Tyr50 [B26]	Cys
Arg89 [Cpep+2]	Cys
Gly90 [A1]	Cys
Ser98 [A9]	Cys
Ser101 [A12]	Cys
Tyr103 [A14]	Cys
Tyr108 [A19]	Cys
***C) neonatal non-Cys-related mutations***	
His29 [B5]	Asp, Gln
Leu30 [B6]	Pro, Gln, Val, Arg
Gly32 [B8]	Ser, Arg, Val
Leu35 [B11]	Pro, Gln
Leu39 [B15]	Pro, Val
[B15-B16]del	His
Val42 [B18]	Gly
Gly47 [B23]	Val
Leu105 [A16]	Pro
Tyr108 [A19]	Asp or Stop
***D) childhood or MODY mutations***	
His29 [B5]	Tyr
Leu30 [B6]	Met
Val42 [B18]	Ala
Gly44 [B20]	Arg
Arg46 [B22]	Gln
Gly47 [B23]	Asp
Phe48 [B24]	Ser
Glu93 [A4]	Lys

a Residue numbers refer to preproinsulin; positions in the mature A- and B chains are given in brackets.

b References are given in **Table S1**.

c Cys insertions have also been observed in the signal sequence and C domain (see **Table S1**).

### Studies of Insulin Biosynthesis

Essential background is provided by the molecular biology of the insulin gene (53–57). In brief, *INS* encodes a single-chain precursor polypeptide, designated *preproinsulin*. Its signal peptide is cleaved on ER translocation. The translocated polypeptide (reduced proinsulin) contains a C domain between B- and A domains (thus connecting Thr^B30^ to Gly^A1^) (58). Folding in the ER requires specific pairing of three disulfide bridges (cystines B7-A7, B19-A20 and A6-A11). These bridges (gold spheres in **Figure 1B**) stabilize the protein and its receptor-binding surface (31, 59–67). Heteronuclear NMR studies of proinsulin (as an engineered monomer) have defined a folded insulin core with flexible C domain (48) as suggested by earlier studies (68–73). The contribution of each disulfide bridge to structure, stability, and activity have been extensively investigated (31, 59–61, 63–67). Together, these bridges provide both interior struts (cystines B19-A20 and A6-A11) and an external staple between chains (A7-B7). Mispairing of insulin’s cysteines (to form disulfide isomers) markedly impairs stability and activity (74–76).

Processing of proinsulin by prohormone convertases (PC1/3 and PC2) liberates the mature hormone (3, 77). Such conversion, occurring in the Golgi apparatus (GA) and immature secretory granules (78), ensures hormonal activity as proinsulin binds more weakly than insulin to the insulin receptor (IR) (79). Insulin and C-peptide are stored within glucose-regulated secretory granules (80) with microcrystalline assembly of Zn^2+^-stabilized hexamers (81–83). Evolution of such assembly foreshadowed its pharmacologic exploitation in clinical formulations (84). The marked susceptibility of the Zn^2+^-free insulin monomer (the active form of the hormone) to fibrillation complicated its manufacture and clinical use in the immediate decades after its discovery in 1921, thus recapitulating evolutionary constraints faced by β-cells due to the implicit threat of toxic misfolding (34, 85). This perspective has been reinforced by studies of a mouse model lacking the β-cell zinc transporter (86). Although key to the stable pharmaceutical formulation of “first-generation” rapid-acting insulin analogs [*lispro* and *aspart* (87), otherwise exhibiting heightened susceptibility to fibrillation (88)], in β-cells such assembly occurs only after exit from the ER and so cannot mitigate toxic misfolding of proinsulin variants.

Unlike native biosynthesis, chemical synthesis of insulin has traditionally employed isolated A- and B-chain peptides (89). The success of insulin chain combination implies that chemical information required for folding is contained within A- and B-chain sequences (90, 91). Hundreds of analogues have been prepared by this protocol, facilitating pharmaceutical innovation (87, 92). Despite the general robustness of insulin chain combination, synthesis of certain analogues has been confounded by low yields (30, 93–99). In selected cases such limitations have been overcome through the use of proinsulin or foreshortened single-chain synthetic intermediates [“mini-proinsulins” (100–103)]. Chemical protein synthesis *via* native ligation of peptide segments (104, 105) has also enabled synthetic access to the proinsulin molecule (106). In addition to their practical utility, such synthetic advances promise to provide insight into structural mechanisms of disulfide pairing (31, 95, 107–109). Sites of mutation among MIDY patients in large measure coincide with past difficulties in synthetic efforts.

### Oxidative Folding Mechanisms

An historic foundation for studies of MIDY mutations in proinsulin has been provided by basic studies of protein folding over the past sixty years. Whereas studies of isolated peptides motifs and model globular domains were often designed to circumvent the complexity of disulfide pairing (28, 29, 110), oxidative protein folding has provided an attractive opportunity to define intermediates investigated by chemical trapping of partial folds (111). An extensive literature pertains to such disulfide-rich globular proteins as bovine pancreatic trypsin inhibitor (44, 112–114), hen egg white lysozyme (115–118) and α-lactalbumin (119–122). Insights from these model proteins and their application to proinsulin and homologous polypeptides underlie efforts to interpret INS mutations associated with toxic misfolding.

**Figure 2:**
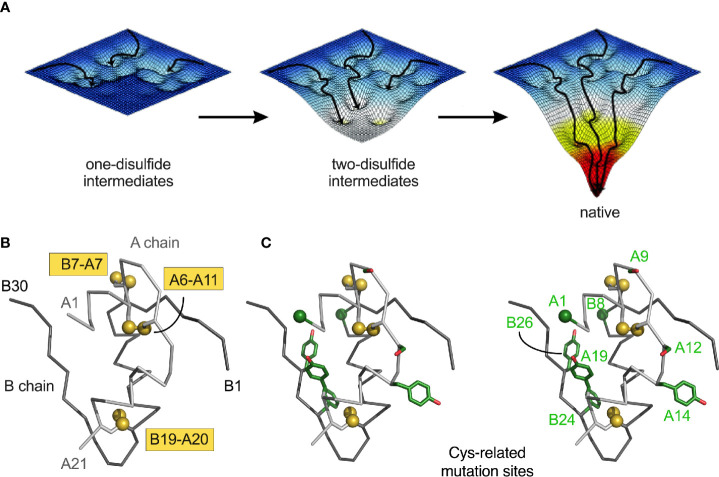
Energy landscape paradigm. **(A)** Landscape maturation: successive disulfide pairing enables a sequence of folding trajectories on ever-steeper funnel-shaped free-energy landscapes. **(B)** Ribbon model of insulin showing the three native disulfide bonds (yellow boxes). Coordinates were obtained from PDB entry 4INS (47). The A- and B chains are shown in light- and dark gray, respectively. **(C)** Stereo view of insulin with Cys substitutions highlighted in green (**Table 1B**). Side chains are shown as sticks; Cys-related sulfur atom and alpha-carbons of Gly^A1^ and Gly^B8^ represented as spheres (one-third Van der Waals radii).

Chemical-trapping studies of proinsulin and homologous proteins have provided evidence for preferential accumulation of one- and two-disulfide intermediates (28, 29, 123, 124). These intermediates define a series of partial folds and corresponding trajectories on successive free-energy landscapes [“landscape maturation”; **Figure 2A**). The landscapes (maturing from shallow to steep; left to right in **Figure 2A**)] are each associated with (a) stepwise stabilization on successive disulfide pairing and (b) a corresponding ensemble of dynamic trajectories constrained by the bridges. Chemical-trapping studies are thus consistent with both multiple folding trajectories on funnel-shaped landscapes and a preferred sequence of specific disulfide intermediates (125) in general accordance with biophysical principles (126–128).

Physiological interpretation of proinsulin refolding studies has been limited by its aggregation near neutral pH [thereby imposing a technical requirement for pH > 9) (29, 129)]. This limitation has been circumvented through the use of mini-proinsulin and IGF-I as more tractable models (28, 29, 59, 62, 108, 110, 123, 130). A structural pathway was proposed based on spectroscopic studies of equilibrium models (31, 59–61, 63–67, 131); this scheme highlights initial formation of cystine A20-B19 within a hydrophobic cluster of conserved side chains between the C-terminal A-chain α-helix and central B-chain α-helix (75, 76, 95). Because in the refolding of mini-proinsulin and IGF-I the A20-B19 disulfide bridge is the first to form (as the only one-disulfide intermediate to accumulate) (28, 29, 110), its pairing defines a biophysical milestone, formation of a specific folding nucleus (31, 131, 132). The predominance of cystine A20-B19 among populated intermediates motivated design of equilibrium models based on pairwise Ala- or Ser substitution of the other cystines (31, 59–67). Such analogues exhibit reduced α-helix content with native-like structure near cystine B19-A20 (31). Mutations in the putative B19-A20-related folding nucleus impair insulin chain combination, biosynthetic expression, and secretion of single-chain precursors in yeast (63, 97, 132–134).

^1^H-NMR spectra of one- and two-disulfide analogues exhibit progressive chemical-shift dispersion with successive disulfide pairing. These data are in accordance with stepwise structural stabilization in the landscape paradigm illustrated above (59, 131). Despite the predominance of A20-B19 pairing as an initial step, folding subsequently proceeds in parallel *via* multiple channels. Mini-proinsulin, for example, can rapidly form cystine A7-B7 or slowly undergo pairing of A6-A11. Although it is not apparent that pairing of cysteines distant in the sequence (such as A7 and B7) should be favored relative to pairing of nearby cysteines (A6 and A11), pairwise substitution of cystine A7-B7 (by Ser) destabilizes insulin more markedly than does pairwise substitution of A6-A11 (132). These findings suggest that nascent structure in the one-disulfide [B19-A21] intermediate either more effectively aligns Cys^A7^ and Cys^B7^ or more significantly impairs pairing of Cys^A6^ and Cys^A11^. These on-pathway two-disulfide intermediate may interconvert with non-native disulfide isomers as off-pathway kinetic traps. The danger posed by such traps has been highlighted in studies of IGF-I and its non-native disulfide isomers (28, 135). Relative isomer stabilities (as probed in a mini-IGF model) are specified by N-terminal residues in the B domain (136, 137). Although the refolding of proinsulin is more stringent, related non-native disulfide isomers (76) may readily be generated by disulfide exchange on addition of a chemical denaturant (75). Corresponding insulin isomers are molten globules whose stability and cooperativity are marginal (76).

Non-native disulfide isomers of proinsulin and related polypeptides have also been observed in transfection studies of mammalian cells (27, 30, 138–140). These studies have exploited electrophoretic mobility differences between native and non-native disulfide isomers in non-denaturing gels [as demonstrated by Arvan, Liu and colleagues (138)]. Less compact structures of non-native states are presumably associated with slower mobilities. Formation of non-native proinsulin isomers has thus been observed on transfection of expression constructs in a variety of mammalian cell lines. Although non-native proinsulin isomers are generally not secreted, mutations can enhance mispairing in the ER (139, 140). Extent of cellular misfolding does not correlate with *in vitro* thermodynamic stability, suggesting that the ER machinery does not evaluate free energies of unfolding (ΔG_u_) as a criterion of quality-control.

Studies of proinsulin variants containing N-terminal substitutions or deletions suggest that contributions of specific side chains to foldability may not be apparent in the native state (141). The substituted side chains may perturb the relative stabilities or kinetic accessibility of disulfide intermediates, for example, disproportionately to effects on the native state, once achieved. Such residues may also contribute to interactions of the nascent polypeptide with ER chaperones and its oxidative machinery (142). Indeed, the ER of β-cells may contain a lineage-specific set of chaperones and foldases required for proinsulin biosynthesis. Defining such a β-cell-specific “ER proteome” defines a key frontier of current research. Cell-type-specific differences in ER proteomes are likely to underlie the inefficient folding and secretion of proinsulin in the majority of human cell lines (143).

Foreshortened “mini-proinsulins” (144) can misfold in yeast to form a metastable disulfide isomer as the predominant secretion product. Such quantitative misfolding indicates that the ER folding machinery of a eukaryotic cell can selectively direct folding into a non-ground-state conformation. Characterizing this alternative pairing scheme and assessing its structural resemblance to the native fold would be of broad interest. Because the aberrant protein is not degraded prior to ER trafficking (*i.e*., it passes ER quality-control checkpoints), such analogues provide models of “stealth” misfolding, in turn leading to secretion of a protein caught in a kinetic trap. As described in the following two sections, clinical mutations in proinsulin conversely exemplify “non-stealth” misfolding leading to activation of the unfolded protein response (UPR) (145–150).

## Monogenic Diabetes and the *INS* Gene

The majority of *INS* mutations cause permanent neonatal-onset DM (**Figure 1** and **Table S1**) (14). Because impaired β-cell function develops prior to maturation of the immune system, the patients present with auto-antigen-negative DM. Similar phenotypes may be caused by mutations in other genes (151), most frequently a heterozygous activating mutation in the β-cell voltage-gated potassium channel (either *KCNJ11* or *ABCC8*, respectively encoding its Kir6.2 and Sur1 subunits) (152, 153). The resulting diabetic phenotype in this genetic background may be transient or permanent. It is important to recognize this subset of neonates or toddlers as in favorable cases they can successfully be treated with oral agents that inhibit the channel (sulfonylureas) rather than by insulin injections (151).

Dominant *INS* mutations are the second most common genetic cause of permanent neonatal DM (7, 13, 14, 16). Such mutations occur in each region of preproinsulin: its signal peptide, B-, C- and A domains (**Table S1**) (9, 10). The majority result in the addition or removal of a cysteine, leading in either case to an odd number of potential pairing sites (**Figure 1A**). Mutations have been found at each of insulin’s six canonical cysteines, generally associated with neonatal onset (**Figure 2B**, **Table 1A**). An additional cysteine may be introduced at various positions in the insulin moiety (**Figure 2C** and **Table 1B**). The resulting odd number of thiol groups leads in general to misfolding and aggregation (11, 12, 18). Even in this context structure may matter, as it is possible that some sites of Cys introduction lead more readily to aberrant intra- or intermolecular disulfide pairing than others, depending on the conformational properties of oxidative folding intermediates and their interactions surfaces. Such biophysical variability would be expected to be associated with differences in ER stress and hence age of DM onset.

Among human MIDY mutations is the same “Akita” substitution (Cys^A7^→Tyr) as in the *Ins2* gene of the *Mody4* mouse (21–23); this dominant murine substitution has thus been characterized as a model of the human syndrome (25–27). The variant murine proinsulin *in vitro* undergoes partial unfolding with increased aggregation (154). Analogous perturbations were found in human insulin- and proinsulin analogues lacking cystine A7-B7 (66, 132). Heterozygous expression of related *Ins2* allele Cys^A6^→Ser in the mouse also causes DM (155).

Identification of identical human and murine mutations at position A7 suggests that the mechanisms of neonatal DM have shared pathogenetic features independent of species (21–23, 25–27). Although β-cell degeneration in the Akita mouse remains incompletely understood, early defects have been observed in the folding and trafficking of both wild-type and variant proinsulins. These defects are associated with elevated markers of ER stress, electron-dense deposits in abnormal ER and GA, and mitochondria swelling—together leading to a progressive decline in β-cell mass (25–27). Evidence for the clinical relevance of these findings has been obtained by the construction of innovative fluorescent proinsulin fusion proteins and their use in cell lines and transgenic mice to detect subcellular localization and aggregation (35–38).

### Deciphering Determinants of Foldability

**Figure 3:**
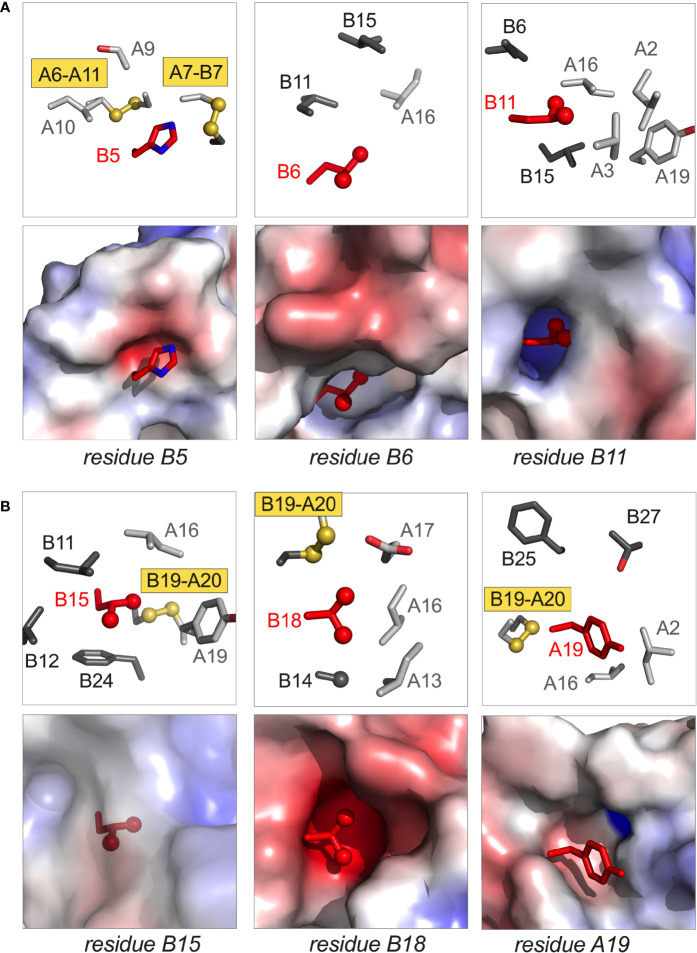
Structural sites of neonatal-onset mutations. **(A)** Spatial environments of residues B5, B6, and B11; **(B)** spatial environments of residues B15, B18 and A19. In each panel the highlighted side chain is shown in red; in each pair of images, stick models are shown in upper panels whereas electrostatic surfaces (calculated in absence of indicated side chain) are shown in the lower panels. In stick models, side chains belonging to the A- and B chains are respectively shown in light and dark gray; Cys-related sulfur atoms (gold) and aliphatic methyl groups (red) are represented as spheres (one-third Van der Waals radii). Coordinates were obtained from PDB entry 4INS (47).

**Figure 4:**
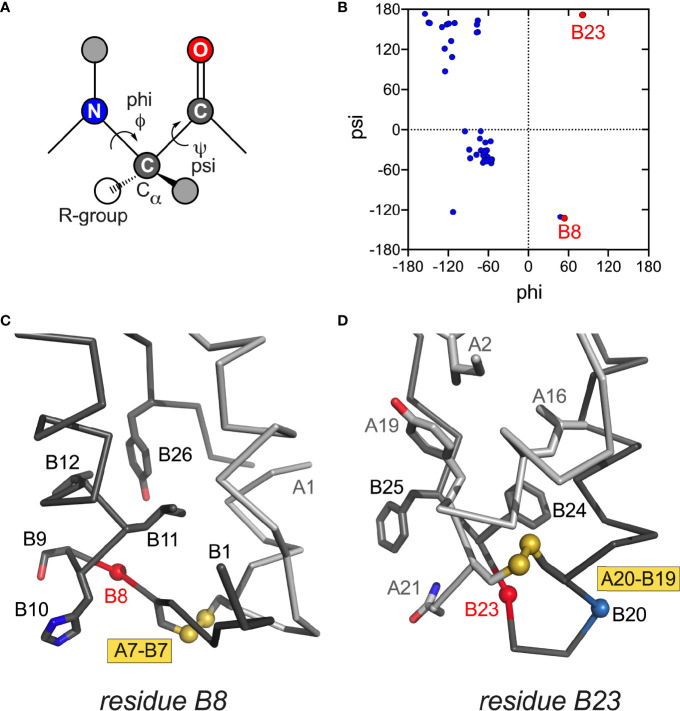
Conformations and structural environments of conserved glycines at positions B8 and B23. **(A)** Main-chain dihedral angles phi (φ) and psi (ψ) in a peptide. **(B)** Ramachandran plot of crystallographic insulin T state (PDB entry 4INS; φ/ψ angles generated using PyMOL and plotted using GraphPad Prism software). Residues B8 and B23 are as labeled (red). In canonical T state Gly^B8^ and Gly^B23^ lie within β-turns with positive φ–angles, thereby residing on the *right* side of Ramachandran plane in regions unfavorable or “forbidden” for L-amino acids. In R state Gly^B8^ residues in α-helix and so on the *left* side of Ramachandran plot (not shown). **(C)** Canonical environment of Gly^B8^ in the T-state β-turn; key nearby side chains are shown. **(D)** Canonical environment of Gly^B23^ in B20-B23 β-turn, shared by T- and R states. Residue B23 is near the side chain of Asn^A21^, and the positive B23 φ angle enables formation of an inter-chain hydrogen bond (A21 side-chain carboxamide NH … O=C B23). Nearby side chains are shown. α-Carbon traces of the A- and B chains are shown in light- and dark gray, respectively. Coordinates were obtained from PDB entry 4INS (47).

**Figure 5:**
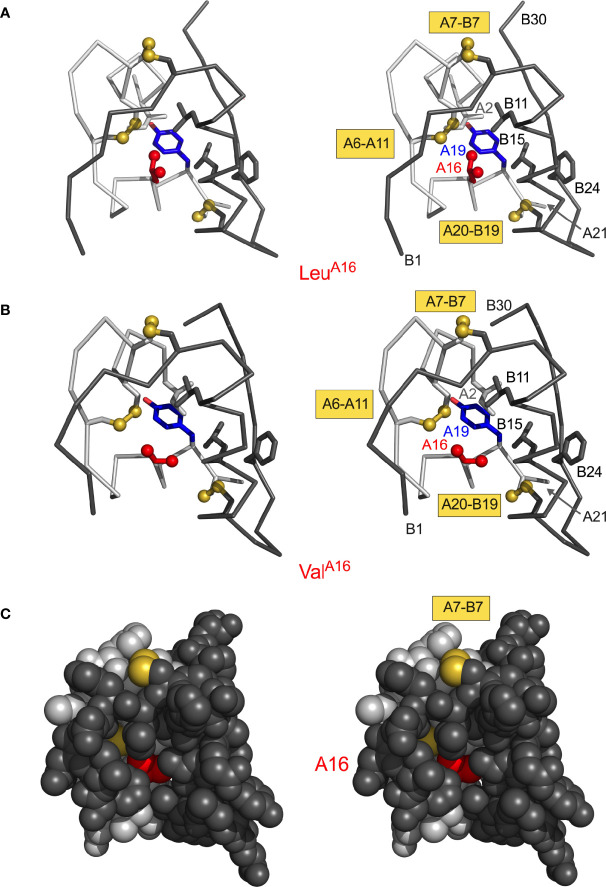
Structural environment of conserved position A16. **(A)** Leu^A16^ packs in core of insulin monomer: ribbon model (stereo pair) showing Leu^A16^ (red) in relation to Tyr^A19^ (blue) and internal side chains Ile^A2^ (light gray), Leu^B11^ (dark gray) and Leu^B15^ (dark gray). A- and B-chain ribbons are shown in light- and dark gray, respectively; disulfide bridges are shown as gold spheres. Molecular coordinates were obtained from PDB entry 4INS (47). **(B)** Corresponding ribbon model (same orientation) of “non-foldable” analogue Val^A16^ (PDB entry 3GKY) (156). Structural similarities highlight cryptic folding defect. **(C)** Stereo space-filling model showing limited exposure of internal Val^A16^ side chain (red) between B-chain surface (dark gray, overlying surface) and A-chain surface (light gray). The solvent-exposed A7–B7 disulfide bridge is shown in gold (top); internal cystine side chains A6–A11 and A20–B19 are not visible.

The Akita variant is representative of a mutant proinsulin with an odd number of cysteines. However, a distinct subset of MIDY- or MODY-associated mutations does not involve cysteine (**Table 1C**). Although widely scattered in the sequence, these mutations occur more often in the B domain than in the A domain—and not at all in the C domain. Because the variant proinsulins retain the six canonical cysteines and yet pair inefficiently, such mutations are of special biophysical interest. A structural overview is provided in **Figures 3**–**5** as described in turn.

Structural relationships in insulin were examined using the monomer derived from a representative wild-type T_6_ zinc insulin hexamer [PDB entry 4INS (47)]. NMR studies have shown that the conformation of an engineered insulin monomer in solution closely resembles the T-state protomer in a zinc insulin hexamer as characterized by X-ray crystallography (60, 157–159). Short- and medium-range NOEs are consistent with spatial relationships in the T state (159). Although positions of C-terminal B-chain residues (B25-B30) are generally less well defined than in crystallographic dimers and hexamers, classical attachment of B24-B28 β-strand to the α-helical core is maintained in solution. The free monomer thus does not exhibit a major change in B-chain conformation [hinge opening of the B20-B23 β-turn (160)]. The present analysis has thus focused on spatial relationships in T-state monomers (as extracted from crystal structures) because of their higher resolution (relative to NMR ensembles) and likely pertinence to proinsulin (48).

*N-terminal segment.* In NMR-derived structures of insulin as a Zn^2+^-free monomer (60, 157, 158), residues B1-B6 are extended (asterisk in **Figure 1B**); B7-B10 comprise a β-turn adjoining the central α-helix. Similar features occur in the crystallographic T-state protomers within Zn^2+^ hexamers (47, 161). The N-terminal five residues favor A7-B7 disulfide pairing *in vitro* (136, 137) and to overall efficiency of proinsulin folding in cell lines (141). These residues are dispensable for receptor binding (162). Although Phe^B1^ has not been identified to date as a site of clinical mutation, studies of *des*-B1 analogues nonetheless suggest that its loose T-state-specific packing against a nonpolar A-chain surface (principally the otherwise exposed side chain of Leu^A13^) contributes to disulfide specification (12). Sites of clinical mutation (His^B5^, Leu^B6^ and Gly^B8^; broadly conserved among vertebrate insulins) have been well characterized (30, 96, 98, 99).

- *Position B5*. In the native state His^B5^ packs within an inter-chain crevice, making one or more hydrogen bonds to carbonyl oxygens in the A chain (**Figure 3A**, left). Clinical mutations are Asp, Gln and Tyr (**Table 1C**); in mammalian cell culture substitution of His^B5^ by Asp blocks the folding and secretion of human proinsulin (30). Although some substitutions impair chain combination (30), Arg^B5^ (found in non-mammalian insulins) is well tolerated. We imagine that His^B5^ and Arg^B5^ form analogous inter-chain hydrogen bonds in the course of disulfide pairing; this hypothesis is in accordance with the respective crystal structures of WT and Arg^B5^-insulin (99). Ala^B5^-insulin (as an engineered monomer) exhibits decreased stability (30), presumably due to the absence of these hydrogen bonds and to a cavity penalty (163, 164). Its solution structure is nonetheless similar to the parent His^B5^ monomer (30), suggesting that critical perturbations in an oxidative folding intermediate can be inapparent in the native state, once reached.
- *Position B6*. Leu^B6^ inserts into a deep inter-chain cavity bounded by the invariant side chains of Leu^B11^, Leu^B15^ and Leu^A16^ (**Figure 3A**, middle). Neonatal-onset mutations are Arg, Gln, Pro and Val (**Table 1C**). Each variant would be expected to be destabilizing in this environment: Arg and Gln *via* insertion of charged or polar functions into a nonpolar cavity, Pro and Val *via* introduction of packing defects. Substitution of the branched and nonpolar side chain of Leu^B6^ by the linear non-polar side chain Met by contrast leads to MODY (**Table 1D**). Delay in clinical onset presumably follows the structural biology: we envision that Met^B6^ can be accommodated within the B6-related cavity but with less optimal packing interactions.
- *Position B8*. Special structural principles pertain to position B8. Neonatal-onset mutations are Arg, Ser and Val (**Table 1C**; also Cys in **Table 1B**). In an insulin or proinsulin monomer in solution (48, 60) Gly^B8^ exhibits a positive ϕ dihedral angle [as in the crystallographic T state (47)] and so occupies a position in the Ramachandran plane ordinarily forbidden to L-amino acids (**Figures 4A, B**). In a protein-folding intermediate an L-amino-acid side chain at B8 would presumably change the orientation of Cys^B7^ and so impair its pairing with Cys^A7^ (**Figure 4C**) (98). The side chain itself would be expected to project into solvent.

Kent, Weiss and colleagues described synthetic studies of human proinsulin variants containing L-Ala or D-Ala at B8 (109). Such protein diastereomers exhibited L-specific impairment of specific disulfide pairing; D-Ala^B8^ was well tolerated, presumably due to its enforcement of a positive ϕ angle favorable to [B7-A7] pairing. These findings corroborated prior studies of mini-proinsulin analogues (134, 165) and insulin chain combination (96). In the latter stereospecific B-chain libraries were exploited to demonstrate that L-substitution at B8 generally impair chain combination whereas yield was generally enhanced by D-substitutions (96). Together, these studies rationalize the invariance of Gly^B8^ among vertebrate insulins and insulin-related polypeptides and the diversity of clinical mutations at this site. Interestingly, Ser^B8^-insulin (but not Ala^B8^-insulin) exhibits substantial biological activity despite its reduced foldability (109). Indeed, its solution structure retain native-like features. Decreased thermodynamic stability was nonetheless observed, presumably due to an unfavorable local main-chain conformation on the right side of the Ramachandran plot (166).

*Central α-helix*. Nascent α-helical structure in the B chain has been observed in one- and two-disulfide analogues containing the key [A20-B19] disulfide bridge (31, 59, 63, 66, 67, 132). Neonatal-onset mutations have been identified at positions B11, B15 and B18 (**Table 1C**) as described in turn.

(i, ii) *Helicogenic residues B11 and B15*. Leu^B11^ and Leu^B15^ each contribute to segmental α-helical propensity (167, 168) and to the nascent clustering of nonpolar residues (31, 131). We imagine that mutations at these sites (Pro or Gln at B11, Pro or Val at B15; **Table 1C**) would impede nascent α-helix formation and in turn initial [B19-A20] disulfide pairing. In the mature structure the B11 side chain is buried within a cavity abutting the nonpolar inner surface of the A chain (**Figure 3A**, right) whereas the B15 side chain packs within a shallower neighboring inter-chain crevice delimited by Cys^B19^ and Phe^B24^ (**Figure 3B**, left). Should native disulfide pairing be achieved, we would expect that that mutations Pro^B11^ and Pro^B15^ would profoundly perturb native structure, stability and self-assembly. Gln^B11^ and Val^B15^ would also be destabilizing, but likely less so than Pro. Gln^B11^ would fit within the B11-related cavity, but its carboxamide group would impose an electrostatic penalty; the smaller, β-branched side chain of Val^B15^ would be predicted to attenuate segmental α-helical propensity (167, 168) and impose a cavity penalty (163).

The importance of Leu^B11^ and Leu^B15^ to folding efficiency was first demonstrated in a model organism. Ala substitutions at these positions (although compatible with α-helix) were found to hinder secretion of mini-proinsulin in *S. cerevisiae* (134). Insulin chain combination was likewise impaired by interchange of Leu^B11^ and Val^B12^, presumably due to perturbed long-range packing (93). Native spacing between Cys^B7^ and Cys^B19^—and hence length of the central B-chain α-helix—are also likely to influence the efficiency of disulfide pairing as a complex MIDY mutation combines a point mutation with deletion with an adjacent residue: Leu^B15^-Tyr^B16^ are replaced by His (43), leaving an even number of residues between the B-chain cysteines.

(iii) *Non-helicogenic residue B18*. Val^B18^ packs near cystine [B19-A20] in a solvent-exposed inter-chain crevice. This environment is polar on one side (due to Glu^A17^) and non-polar on other sides (due to the cystine, Ala^B14^, Leu^A13^ and Leu^A16^). Although the β-branched side chain of Val is not in principle favorable within an α-helix (167, 168), its mutation to Gly (also of low helical propensity; **Table 1C**) would enhance main-chain flexibility and introduce an inter-chain packing defect; each perturbation could reduce efficiency of [B19-A20] disulfide pairing. In the native state ^1^H-^2^H exchange studies in D_2_O have established that the main-chain amide proton of Val^B18^ is the most highly protected site in insulin (159). Extending this to variant on-pathway folding intermediates, we propose that enhanced segmental conformational fluctuations and decreased thermodynamic stability could each contribute to impaired biosynthesis.

*B20-B23 β-Turn*. The B chain contains a U-turn between its central α-helix and C-terminal β-strand (B24-B28). This super-secondary motif requires a solvent-exposed β-turn (Gly-Glu-Arg-Gly tetrapeptide motif). Like Gly^B8^ (above), the flanking glycines each exhibit positive ϕ angles associated with a specific pattern of hydrogen bonds within the turn (47). Discussed more fully in the following section (MODY), mutation of Gly^B23^ to Val is associated with neonatal-onset DM (**Table 1C**). Cell-based and biophysical studies of this mutation have demonstrated profound perturbations (97). Qualitative NMR studies suggest that the β-branched side chain leads to transmitted perturbations in the position or conformation of the following B24-B27 segment (12).

*A-chain mutations*. Studies of peptide models have suggested that initial pairing of cystine [B19-A20] is coupled to nascent α-helical conformations of the A16-A19 segment, coincident with nonlocal hydrophobic collapse of Leu^A16^ and Tyr^A19^ within a folding nucleus (31, 131). Indeed, substitutions at these sites were found to impair the yield of insulin chain combination (94, 95, 156). In accordance with the above mechanism and such synthetic experience, recent clinical studies have uncovered neonatal-onset MIDY mutations Pro^A16^ and Asp^A19^ (**Table 1C**).

The structural environments of α-helical residues A16 and A19 are distinctive. Whereas Tyr^A19^ projects from a non-polar crevice (lined in part by cystine [B19-A20]) to expose its *para*-hydroxyl group (**Figure 3B**, right), the side chain of Leu^A16^ is inaccessible to solvent (**Figure 5**). Asp^A19^ would place a negative charge within the non-polar confines of the core. Pro^A16^ would perturb segmental main-chain conformation and (when modelled in a native-like framework) introduce both side-chain steric clash and a destabilizing cavity. The essential contribution of Leu^A16^ to protein-folding intermediates has been demonstrated through studies of Val^A16^-proinsulin and Val^A16^-insulin (156). Although this substitution is compatible with a native-like crystal structure (essentially identical to WT insulin), Val^A16^ markedly impairs both insulin chain combination and cellular folding of the variant proinsulin (156). Because Val^A16^-insulin also exhibits high biological activity (156), the evolutionary invariance of Leu at this position presumably reflects its cryptic yet key contribution to folding efficiency.

MIDY mutations have not been identified in the N-terminal A-domain α-helix (residues A1-A8). Their absence may simply reflect incomplete sampling of patients to date; however, it is also possible that non-cysteine residues in this segment are tangential in the mechanism of disulfide pairing. Indeed, successful combination of variant A chains containing Gly at positions A1-A2, A1-A4 or A1-A4 (in each case with WT B chain S-sulfonate) provided evidence that an N-terminal A-chain α-helical conformation is not required for native disulfide pairing (95). Such dispensability is in accord with a putative structural pathway in which segmental folding of this α-helix is a late event.

## From MIDY to MODY

*INS* mutations may also be associated with onset of DM in childhood or adolescence (**Table 1D**) (169–171); diagnoses may be carried as auto-antibody-negative presumed Type 1 DM or Type 2 DM. Substitution of Val^B18^ (**Figure 3B**, center) by Ala (172) was identified as a MODY allele (DM onset <25 years of age, autoantigen negative) in a three-generation Italian pedigree (three siblings, the parent and presumed grandfather) (172). Unlike MIDY patients with neonatal onset, birth weights were normal. The Ala mutation at position B18 would be expected to enhance segmental α-helical propensity (167, 168), but introduce a destabilizing cavity (163, 164) adjacent to the critical [B19-A20] disulfide bridge. Unlike the perturbations introduced by Gly^B18^ (above), these effects would offset to yield, rationalizing a mild net impairment of initial disulfide pairing.

**Figure 6:**
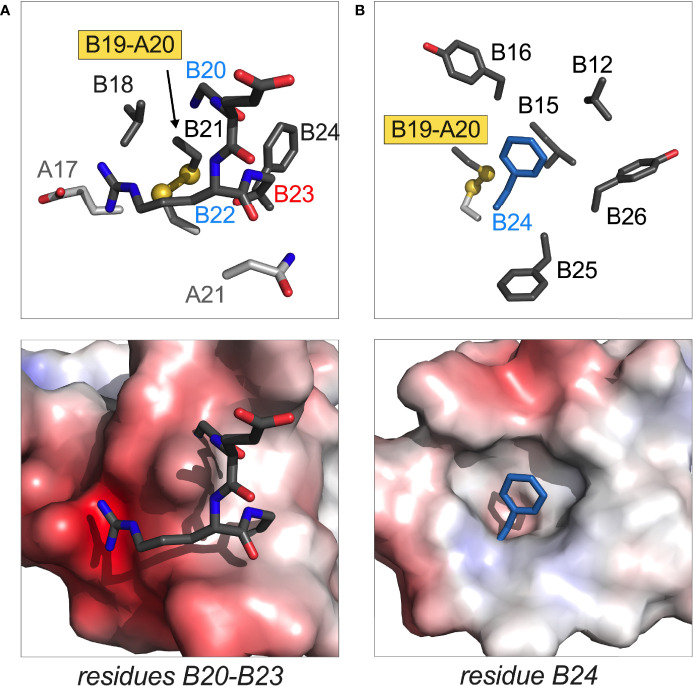
Structural sites of MODY mutations. **(A)** Residues B20 and B22 in B-chain B20-B23 β-turn; **(B)** residue B24 anchoring this β-turn and adjoining B24-B28 β-strand. In each pair of images, stick models are in upper panel and electrostatic surfaces in lower panel. The latter highlights the groove or cavity occupied by the designated structural element; blue and red surfaces are coded by positive or negative electrostatic potential. In stick models main-chain atoms in A- or B main chains are shown in light or dark gray, respectively. Disulfide bridges are shown as balls and sticks with sulfur atoms in gold (one-third Van der Waals radii). The side chain of Phe^B24^ in **(B)** is shown as dark blue stick (not related to electrostatic potential). Coordinates were obtained from PDB entry 4INS (47).

Four additional MODY mutations occur within the B20-B23 β-turn and its aromatic anchor at B24: Gly^B20^→Arg, Arg^B22^→Gln, Gly^B23^→Asp and Phe^B24^→Ser (**Figure 6**). Although the mechanism by which Gln^B22^ causes MODY is not apparent, L-amino-acid substitutions of Gly^B20^ or Gly^B23^ would be expected to alter their respective ϕ dihedral angles. It has previously been reported that Ala substitutions impair the expression of mini-proinsulin in *S. cerevisiae* and impede chain combination, whereas efficient disulfide pairing *in vitro* can be rescued by D-Ala substitutions (97). That B23 mutations may cause either neonatal onset (Val^B23^) or delayed onset (Asp^B23^) suggests that details of side-chain chemistry influence folding efficiency.

Ser^B24^ (originally designated insulin *Los Angeles*) is associated with variable genetic penetrance with hyperinsulinemia. The latter finding indicates that Ser^B24^-proinsulin can in fact fold in the β-cell ER, undergo proper trafficking and processing to mature Ser^B24^-insulin (173). In cell culture the variant proinsulin nonetheless induces ER stress, albeit at a level below MIDY variants (12). In vivo mutational induction of mild or moderate ER stress can presumably cause (depending on other genetic risk alleles and environmental factors) slow but progressive loss of β-cell mass (174, 175) as in the Akita mouse (24, 176).

**Figure 7:**
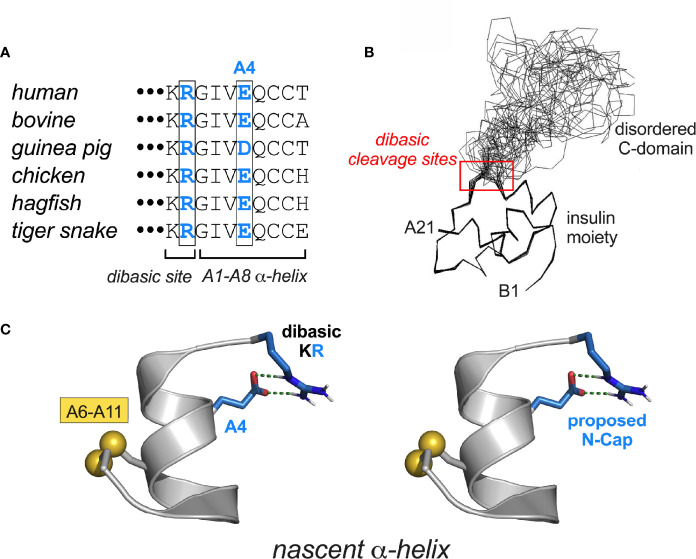
MIDY-related mutations at CA junction. **(A)** Vertebrate sequence alignment showing conserved KR dibasic site (CA junction) and acidic side chain at residue A4. **(B)** Solution structure of proinsulin (line drawing) showing the folded insulin moiety and disordered C-domain (48). The dibasic RR (BC junction) and KR sites (CA junction) are within red box. **(C)** Proposed stabilization of a nascent α-helix in proinsulin folding intermediate by junctional (*i*, *i*+4) salt bridge between residues Arg and Glu^A4^ (blue in panels **A**, **C**). The putative salt bridge was modeled as an α-helical N-Cap element (177) using PyMOL.

The final MODY-associated mutation occurs on the surface of the A domain: Glu^A4^→Lys (**Table 1D** and **Figure 7A**). That this substitution should perturb the folding of proinsulin seems surprising given (a) the absence of structural constraints at this position in insulin and (b) the broad tolerance of insulin chain combination to substitutions within the N-terminal A-chain α-helix (95). We speculate that Lys^A4^ introduces a subtle perturbation in proinsulin through electrostatic repulsion of the dibasic element at the CA junction (red box in **Figure 7B**). In particular, nascent α-helical structure in the A1-A8 segment may be stabilized by a salt bridge between “Arg^A0^” (*i.e*., the final residue of the C domain [position 89 of preproinsulin]; **Figure 7A**) and WT Glu^A4^ (**Figure 7C**). Such an interaction, together with Gly^A1^, could in essence provide a favorable N-Cap (177), which could overcome the adverse helical propensities of the three β-branched residues in this segment (Ile^A2^, Val^A3^ and Thr^A8^). This contribution would not pertain to insulin chain combination due to the absence of Arg^A0^ (an analogous C-capping salt bridge from Glu^A4^ to the A1 α-amino group would be blocked by its deprotonation at the reaction pH of 10.5).

## Diversity of INS-Related Disease Mechanisms

For completeness, we note that mutations in the insulin gene that are not associated with impaired folding can nonetheless be associated with adult-onset DM phenotypes of variable penetrance (57) (**Table S1**). Such heterogeneity is in accord with “Murphy’s Law of genetics”: in a complex pathway or set of mechanisms, what can go wrong will go wrong. For example, insulin variants *Wakayama* and *Chicago* (*i.e*., classical insulinopathies Val^A3^→Leu and Phe^B25^→Leu respectively) markedly impede receptor binding (173) in association with mutant hyperinsulinemia (178). These mutations directly perturb the hormone-receptor interface (160). A complementary example is provided by diabetes-associated mutation His^B10^→Asp, which enhances receptor binding (179). Although Asp^B10^ would introduce a favorable electrostatic interaction at the hormone-receptor interface, in β-cells Asp^B10^-proinsulin exhibits inappropriate sorting to a constitutive granule (180, 181). Unlike glucose-regulated secretory granules, constitutive granules lack prohormone convertases, and so the patients exhibit mutant hyperproinsulinemia. Yet another syndrome is characterized by impaired prohormone processing leading to circulation of a split proinsulin with reduced activity (182).

## Evolution at the Edge of Foldability

**Figure 8:**
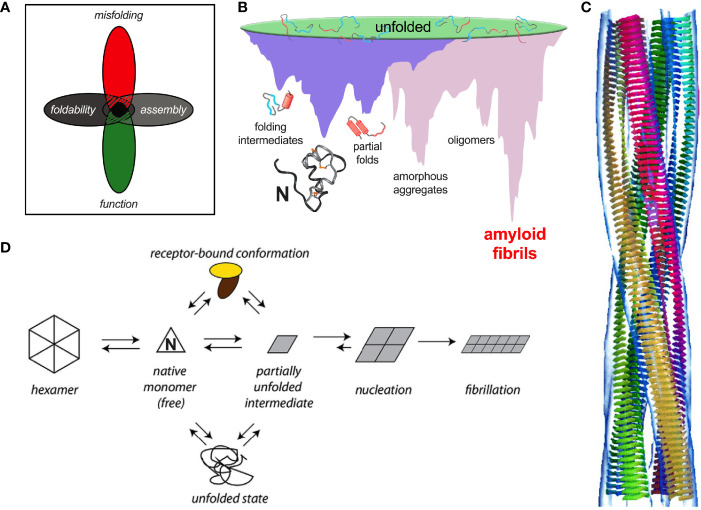
Evolutionary constraints and insulin fibrillation. **(A)** Venn diagram showing intersection of multiple constraints: function, foldability, misfolding, and assembly (183). **(B)** Energy landscape of protein folding (purple) and coupled landscape of aggregation (pink) (184). Cytotoxic oligomers may form as off-pathway intermediates en route to amyloid. **(C)** Models of protofilament packing based on low-resolution cryo-EM images. The image is reproduced from the reference (185), Copyright (2002) National Academy of Sciences. **(D)** General scheme of insulin fibrillation *via* a partially unfolded monomeric intermediate (parallelogram at center) (186). The native state (triangle) is protected by classic self-assembly (far left). Disassembly leads to an equilibrium between native and partially folded monomers. The receptor-bound conformation of insulin (top) may also participate in this equilibrium. This partial fold may unfold completely (bottom) as an off-pathway event or aggregate to form an amyloidogenic nucleus en route to a proto-filament (right).

Protein evolution is generally enjoined by overlapping biological constraints, including biosynthesis, structure, and function (**Figure 8A**). Particular residues in insulin may thus contribute to one or more critical mechanisms, including nascent foldability in the ER, protection from intra- or extracellular toxic misfolding, trafficking from the ER through the GA to glucose-regulated secretory granules, self-assembly within these granules, disassembly of Zn^2+^-insulin hexamer in the portal circulation and in turn receptor binding. The stringency of these overlapping constraints rationalizes the limited sequence variation among vertebrate insulins (47).

Evolutionary constraints may be coincident or opposing at a given position. An example of a coincident constraint is the concurrent contributions of invariant Phe^B24^ to core packing, dimerization and receptor binding. Opposing constraints call for compromise. An example is provided by Gly^B8^, invariant as an achiral amino acid free to roam in the Ramachandran plane. Systematic studies of L- or D substitutions have suggested that at B8 kinetic determinants of foldability are at odds with conformational requirements of receptor binding (96, 98). Whereas a positive ϕ dihedral angle (enforced by a D-substitution) facilitates disulfide pairing, a D side chain impedes receptor binding. Conversely, negative dihedral angle (like that of an L-amino acid) impair folding efficiency but may be compatible with receptor binding (96, 98). These opposing requirements presumably underlie the invariance of glycine – the only achiral amino acid – at a site of conformational change. A switch in conformation of Gly^B8^ between the right side of the Ramachandran plot and the left (respectively corresponding to positive or negative ϕ angles) was anticipated by the classical TR transition among zinc insulin hexamers (187). Although such allostery may pertain only to hexamers (99), the TR transition exemplified the long-range transmission of conformational change (188) —a theme central to the transmembrane propagation of an insulin signal *via* receptor reorganization (189–191). The examples posed by clinical mutations at B24 and B8 (**Table 1C**) suggest that premature adoption of the hormone’s receptor-engaged conformation within β-cells (either by proinsulin in the ER or GA or by insulin in secretory granules) may trigger toxic misfolding.

Recent co-crystal and cryo-EM-derived structures of insulin bound to receptor fragments have demonstrated the function of a protective hinge in B chain (160, 189, 190, 192). Mechanisms of hormone-receptor recognition [for review, see (191)], extend to IGF-I as visualized in a landmark series of homologous cryo-EM-derived structures of respective receptor ectodomain complexes (189, 190, 193–195). As predicted based on studies of “anomalous” insulin analogues (157, 196, 197), detachment of the C-terminal β-strand (residues B24-B28) enables both its own binding in a groove between receptor elements L1 and αCT (respectively at the N- and C-terminal ends of the IR α-subunit); the latter element also packs against the N-terminal A-chain α-helix.

Insulin’s B-chain hinge—opened on receptor binding—may represent an evolutionary response to the danger of proteotoxicity. This danger, aggravated by exposure of non-polar surfaces, is intrinsic to the coupled folding/misfolding landscapes wherein the true ground state is defined by β-sheet-rich amyloid (**Figure 8B**). Models of insulin amyloid as superhelices of protofilaments have been derived at low resolution by cryo-EM (**Figure 8C**). Studies of insulin fibrils by infrared and Raman spectroscopy have demonstrated a predominance of β-sheet (198–200) in accordance with fibril X-ray diffraction (201–203). Despite the striking biophysical features of fibrils as a universal thermodynamic ground state of polypeptides (**Figure 8B**) (85), oligomeric intermediates in the pathway of fibrillation pose the greater cytopathic danger (**Figure 8D**) (204).

Recent evolutionary studies of insulin have highlighted the importance of Phe^B24^, whose conserved aromatic ring plays multiple roles: anchoring the native B-chain β-strand, stabilizing the α-helical core, and contributing to both self-assembly (47) and hinge opening on receptor binding (197). In the open state the aromatic ring binds within a classical nonpolar pocket at the hormone-receptor interface (189, 190, 195). On substitution of Phe^B24^ by Gly, native function is paradoxically retained (157). Comparative studies of “register shift” analogues indicate that an alternative mode of receptor binding supervenes in which Phe^B25^ takes the place of the missing Phe^B24^ (52); residues B20-B24 form a flexible pentaloop rather than an aromatic-anchored β-turn (205). This alternative binding mode is apparently disallowed in evolution due to toxic misfolding of Gly^B24^-proinsulin (as evidenced impaired folding efficiency, induction of ER stress and impaired secretability in transfected cell models) and possibly by the heightened susceptibility of Gly^B24^-insulin analogues to fibrillation (52). Evidence for the paradoxical evolution of vertebrate insulins to the edge of foldability has been provided by biophysical studies of a native-like variant, Tyr^B24^-insulin (40). Although providing the C-terminal B-chain β-turn and β-strand with an homologous “aromatic anchor,” Tyr^B24^ is also disallowed due its perturbation of biosynthesis and induction of ER stress. Indeed, of the 20 natural amino acids, only Phe at position B24 enables the efficient biosynthesis of proinsulin (40). We speculate that such marked sensitivity to mutation—signifying the paradoxical non-robustness of an adaptive landscape (40)—will be found at many or most sites associated with neonatal-onset DM (**Table 1C**).

Because, to our knowledge, clinical mutations that selectively perturb insulin’s hexameric structure and storage in secretory granules have not been described, this review has not focused on these processes. Any such perturbations would be downstream from the major sites of perturbation in the MIDY syndrome: misfolding in the ER and impaired trafficking through the GA. It is possible, however, that processes in the secretory granule are affected by Ser^B24^ and Asp^B10^ in concert with other perturbations. (i) *Phe^B24^→Ser.* The invariant aromatic ring of Phe^B24^ packs at the dimer interface. Its substitution by Ser^B24^ impairs self-assembly (as monitored by gel-filtration) and leads to accelerated disassembly of the R_6_ hexamer once formed (40). Receptor binding and biological activity are low. (ii) *His^B10^→Asp*. The conserved imidazole ring of His^B10^ coordinates the axial zinc ions at the trimer interface of insulin hexamers (47). Genetic variant Asp^B10^ causes a diabetes syndrome characterized by baseline mutant proinsulinemia due to constitutive secretion (180) as the mutation perturbs specific trafficking to glucose-regulated secretory granules (34, 57). The corresponding substitution in insulin blocks both zinc binding and trimer formation *in vitro* (206, 207). Asp^B10^-insulin exhibits increased affinity for both IR and IGF-1R with prolonged residence times in association with augmented mitogenic signaling (179, 208–211).

## An Evolutionary Hypothesis

**Figure 9:**
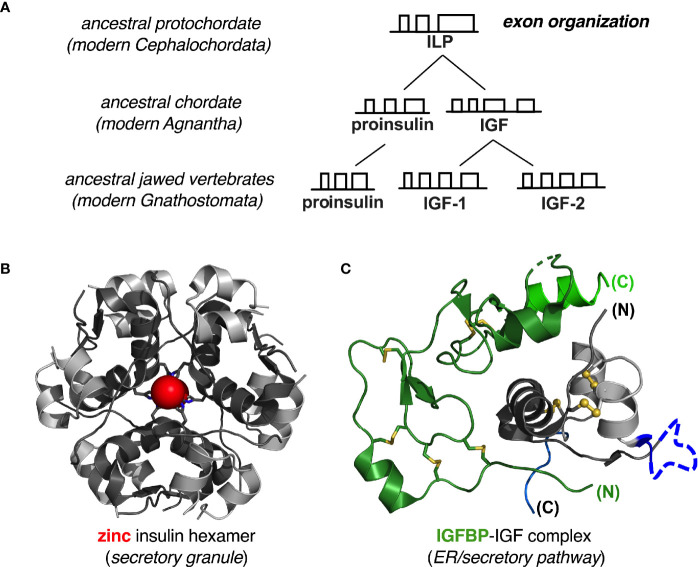
Evolution of insulin-like genes in vertebrates. **(A)** Steiner and colleagues proposed that a primordial insulin-like gene in protochordates was the common ancestor of vertebrate insulin/IGF factors (215, 217). Amphioxus ILP represents this ancestral gene (as a “living fossil”) prior to gene duplication in early agnathan vertebrates. Further gene duplication results in distinct IGF-I and IGF-II genes found in all gnathosomes (jawed vertebrates). Independent gene duplication events occurred during the evolution of invertebrate insulin-like peptides (not shown). See **Supplemental Figure S1A** for further details of exonic structure. **(B)** Proinsulin folds autonomously, and the mature hormone is stabilized by native self-assembly: ribbon model of classical zinc-insulin hexamer. The A- and B chains are shown in light- and dark gray, respectively; the two overlying axial zinc ions are shown as red spheres (enlarged for emphasis at twice Van der Waals radii), each coordinated by three His^B10^ side chains (sticks). **(C)** IGF-I and IGF-II fold together with specific IGF binding proteins: ribbon model of illustrative IGFBP-IGF complex. We hypothesize that heterodimeric folding “rescues” the foldability of the IGFs, whose folding properties would otherwise be ambiguous. Color codes as follows: IGF domain A (light gray), domain B (dark gray), domain C (dark blue) and domain D (light blue); IGFBP N-terminal domain (dark green) and C- terminal domain (light green). Molecular coordinates were respectively obtained from PDB entries 1MSO (zinc insulin hexamer) (186) and 2DSP (IGF-1/IGFBP-4 complex) (219).

Given the ancestral history of metazoan insulin-like proteins over the past 540 million years and its broad radiation among diverse body plans (212–214), why might vertebrate proinsulins be susceptible to misfolding and lacking in mutational robustness? A possible answer is given by the history of the *INS* gene as traced by the late D.F. Steiner and coworkers (215–218). This seminal study characterized an insulin-like gene encoding an insulin-like protein (ILP) in an extant protochordate (amphioxus; *Branchiostoma californiensis*) (**Figure 9A**). The predicted polypeptide precursor pro-ILP contains a C-terminal peptide resembling the D and E domains of vertebrate IGFs, suggesting an intermediate form linking the ancestral proto-insulin gene with modern IGF genes. In accordance with this perspective, *ILP* is the only *INS*-like gene in amphioxus; its genome also contains a single gene encoding a putative insulin-IGF receptor (216) and a single gene encoding a putative IGF-binding protein (IGFBP) (220, 221).

ILP was thus proposed to combine the functions of insulin, IGF-I and IGF-II prior to the duplication the proto-insulin gene and specialization of distinct factors (215). Evolutionary changes in intron-exon structures are shown in greater detail in **Supplemental Figure S1A**. In this scheme conversion of a metazoan proto-insulin gene to ILP would have been effected by a nonsense-to-sense mutation at the end of the A-domain-encoding sequence in the putative proto-insulin gene; conversion of ILP to proto-lGF would have been effected by an upstream shift in the intron donor site into the B-domain-encoding exon. IGF-I and -II genes subsequently evolved from the posited proto-IGF by insertion of an intron into the E-encoding domain followed by gene duplication.

Thus, predating the divergence of insulin and IGFs as distinct gene products, ILP retains framework residues conserved among vertebrate insulins and IGFs, including the six canonical cysteines, Leu^B6^, Leu^B11^, Leu^B15^, Val^B18^, Leu^A16^ and Tyr^A19^—hotspots for MIDY mutations (**Table 1C**). Whereas mammalian insulins contain Leu^B17^, however, residue B17 in ILP is Phe as in IGFs. Similarly, ILP residue A8 is Tyr, resembling the homologous Phe in IGF-I and IGF-II but unlike Ala^A8^ or Thr^A8^ in mammalian insulins (215, 218). ILP would not be expected to undergo insulin-like self-assembly: (a) it lacks a His at position B10 and so would not be expected to coordinate zinc ions; and (b) dimerization would be predicted to be impaired by ILP residues Ala^B12^ and Ser^B26^ (in place of Val^B12^ and Tyr^B26^) (183, 222). Representative vertebrate insulin B-chain sequences and IGF-I B-domain sequences are shown in **Supplemental Figure S1B**.

Given the evolutionary framework established by Steiner and coworkers (215–217), we hypothesize that the primordial insulin/IGF precursor protein folded as a heterodimer in partnership with a proto-IGFBP. Such heterodimeric folding occurs in vertebrate IGF-IGFBP systems (221, 223–226) and appears to compensate for the ambiguous refolding properties of IGFs *in vitro* (28, 29, 123, 130). We envisage that in heterodimeric folding requirements of foldability are relaxed in each partner (when considered in isolation). Crystal structures of human IGF-I/IGFBP complexes [illustrated in a representative case in **Figure 9C** (219)] exhibit extensive engagement of IGF surfaces adjoining disulfide bridges and sites of MIDY mutations. This model predicts that the foldability of pro-IGF variants in mammalian cells would be more robust to MIDY-like mutations than is proinsulin—*but only in cells co-expressing one or more IGFBPs*.

Proinsulin by contrast folds in the ER as an autonomous monomer, aided by chaperonins and oxidoreductases but not, to our knowledge, by specific proinsulin-binding proteins. Native zinc-mediated self-assembly of insulin (**Figure 9B**) can include proinsulin [which can form corresponding hexamers (69)], but such self-assembly occurs in secretory granules and not in the zinc-poor environment of the ER. Although IGFBPs do not bind to insulin or proinsulin, this model predicts that engineered proinsulin-binding proteins may be designed to enhance the foldability of WT and variant proinsulins. Although such artificial proteins are unlikely to find therapeutic application, they may be of interest as reagents to probe the mechanism of proinsulin folding *in vivo*, including steps susceptible to misfolding.

## Structural Determinants of ER Quality Control

Arvan and colleagues have studied the ER folding of proinsulin in β-cell lines using a systematic set of variants that can form only one or two disulfide bonds; to this end, specific disulfide bridges were removed by pairwise mutagenesis (227). These constructs differed in biosynthetic properties and so provided probes of quality-control determinants. Their results demonstrated that cystines A20-B19 and A7-B7 (but not cystine A6-A11) are critical to enable native folding and ER exit. Prior biophysical studies of an insulin analogue lacking cystine A7-B7 (due to pairwise Ser substitution) demonstrated a more marked decrease in stability and chain-combination efficiency relative to analogous analogues lacking A6-A11 (60, 132). Further studies on single-chain insulin analogues (67) and IGF-1-related peptides and peptide fragments (29, 31, 60) provided evidence for a kinetic pathway in which pairing of cystine A20-B19 provides a required first step to stabilize a native-like molten mini-core (31). Native-like NOEs were observed in such a one-disulfide peptide model even in the absence of stable secondary structure (31). Together, these cell-based and *in vitro* studies suggest possible structural features that might be sensed by ER quality control: as a general principle, the more destabilizing the disulfide intermediate or isomer destabilizing, the greater the degree of exposed non-polar surfaces and in turn the intervention of detection and degradation by the quality-control machinery.

Whereas variant proinsulin polypeptides without interdomain disulfide bridges cannot be secreted (227), non-native disulfide isomers can accumulate and evade ER quality control (138, 140). Early work from the Arvan laboratory demonstrated secretion of mispaired disulfide isomers in cells using various single-chain insulin constructs (138, 140). Indeed, prior studies of IGF-I revealed that its oxidative refolding *in vitro* yielded two isoenergetic products (28, 123). Although these had similar α-helical propensities and thermodynamic stabilities, 2D ^1^H-NMR spectra were remarkable for distinct well-dispersed patterns of chemical shifts, indicative of different three-dimensional structures (28). Unlike IGF-I and its disulfide “swapped” isomer, insulin disulfide isomers are less stable and less well-ordered than is native insulin (75, 76). The respective N-terminal segments of proinsulin and IGF-I contribute to such salient differences in the fidelity of disulfide specification and relative stability (228, 229). The chain asymmetry of non-Cys-related MIDY mutations—more in the B domain than in the A domain (**Figure 1**)—is consistent with a hierarchical disulfide pathway in which nascent structure in the B domain provides a structural template for folding of the A domain (95).

Mutations that impair the foldability of proinsulin (or efficiency of insulin chain combination (95) can nonetheless be compatible with high activity (108). This lack of correlation suggests that determinants of quality control in the ER differ from determinants of receptor binding. A prominent example is provided by substitution of invariant Leu^A16^ by Val (156). This cavity-associated mutation (not [yet] seen among MIDY patients) markedly impairs both cellular folding of Val^A16^-proinsulin and chain combination, and yet substantial biological activity is retained once the native state is reached (94, 156). Similarly, folding of Ser^B8^-proinsulin is significantly reduced *in vitro*, yet IR affinity is similar to WT insulin (109). A recent study reported that substitution of Phe^B24^ by Tyr (also not [yet] seen among MIDY patients) blocks cellular folding (40) whereas the corresponding two-chain insulin analogue retains substantial activity in a rat model of DM.

## Concluding Remarks

We imagine that insulin’s conserved side chains, as exemplified by Phe^B24^, play different roles in the course of a complex conformational “life cycle.” If so, this would represent a marked compression of structural information within a short protein sequence. The present cryo-EM revolution promises to provide snapshots of structures through this life cycle, likely to be extended by solid-state NMR-based models of non-native insulin aggregates and fibrils. As we celebrate the Centennial of insulin’s discovery in Toronto in 1921 (1, 230)—and coincidentally the gold anniversary of its high-resolution crystal structure at Oxford in 1971 (161)—it is remarkable to appreciate how much remains to be discovered in relation to biosynthesis, folding, function and evolution. Further, in a related review article, J. S. Flier and C. R. Kahn have discussed how the discovery of insulin has defined a milestone in the history of molecular medicine that extends beyond the insulin molecule itself (231).

This review has focused on the structural lessons of the mutant proinsulin syndrome (4–6). Patient-derived experiments of nature are providing an opportunity to investigate biophysical principles at the intersection of cell biology and human genetics. As envisioned by classical diffusion-collision and framework models (232), folding of globular proteins (such as proinsulin) represent the coalescence of discrete subdomains (233, 234). Even as funnel-like energy landscapes make possible parallel events in folding (126), the existence of preferred trajectories (235) is implied by disulfide trapping studies of insulin-related polypeptides. Biophysical studies of these trajectories and equilibrium models promise to deepen a structural understanding of MIDY/MODY mutations. Sites of mutation reflect mechanisms of folding or misfolding that may not be apparent in the native state (9, 10, 19, 51). Many of the principles discussed here were foreshadowed in pioneering efforts toward the total chemical synthesis of insulin wherein specific disulfide pairing posed a key challenge to chain combination (89).

Foldability is an evolved property (236), highlighting the general threat of toxic misfolding as a hidden constraint in protein evolution. In the genetics of proteotoxic diseases these principles connect bench to bedside. Critical questions for continuing investigation include: can over-expression of the WT *INS* gene in response to peripheral insulin resistance likewise tax the folding capacity of the β-cell and induce ER stress analogous to that of the mutant proinsulin syndrome? Might structural mechanisms of misfolding due to MIDY mutations broadly inform a hidden landscape of toxic aggregation awaiting WT biosynthesis? A key frontier in molecular metabolism is thus defined by the role of the UPR and chronic ER stress in the progression of non-canonical Type 2 DM (9, 24, 25). Molecular dissection of how β-cells respond to the challenge of proinsulin overexpression (237) is of compelling translational interest as a strategy to arrest the progression of prediabetes to frank diabetes (9, 38, 238). Structural lessons of the mutant proinsulin syndrome may thus inform UPR-based approaches to mitigate the growing pandemic of diabesity.

## Author Contributions

MW oversaw preparation of the manuscript and wrote the first draft. All authors contributed to the article and approved the submitted version.

## Conflict of Interest

The authors declare that the research was conducted in the absence of any commercial or financial relationships that could be construed as a potential conflict of interest.

## Publisher’s Note

All claims expressed in this article are solely those of the authors and do not necessarily represent those of their affiliated organizations, or those of the publisher, the editors and the reviewers. Any product that may be evaluated in this article, or claim that may be made by its manufacturer, is not guaranteed or endorsed by the publisher.

## Abbreviations

DM, diabetes mellitus; ER, endoplasmic reticulum; GA, Golgi apparatus; MIDY, mutant *INS*-gene-induced diabetes of youth; MODY, maturity-onset diabetes of the young, and NMR, nuclear magnetic resonance. Amino acids are designated by standard one- and three-letter codes.
