# Supplementary material for: Structural Lessons From the Mutant Proinsulin Syndrome
Source: Front Endocrinol (Lausanne). 2021 Sep 30;12:754693. doi: 10.3389/fendo.2021.754693 (PMC8514764; doi:10.3389/fendo.2021.754693)
Supplement: Supplementary file 1 [file DataSheet_1.pdf]

SUPPLEMENTAL INFORMATION

for

Structural Lessons from the Mutant Proinsulin Syndrome

Balamurugan Dhayalan, Deepak Chatterjee, Yen-Shan Chen, & Michael A. Weiss*

Department of Biochemistry and Molecular Biology, Indiana University School of Medicine, Indianapolis IN 46202 USA

*Address correspondence weissma@iu.edu

SUPPLEMENTAL FIGURE S1

A

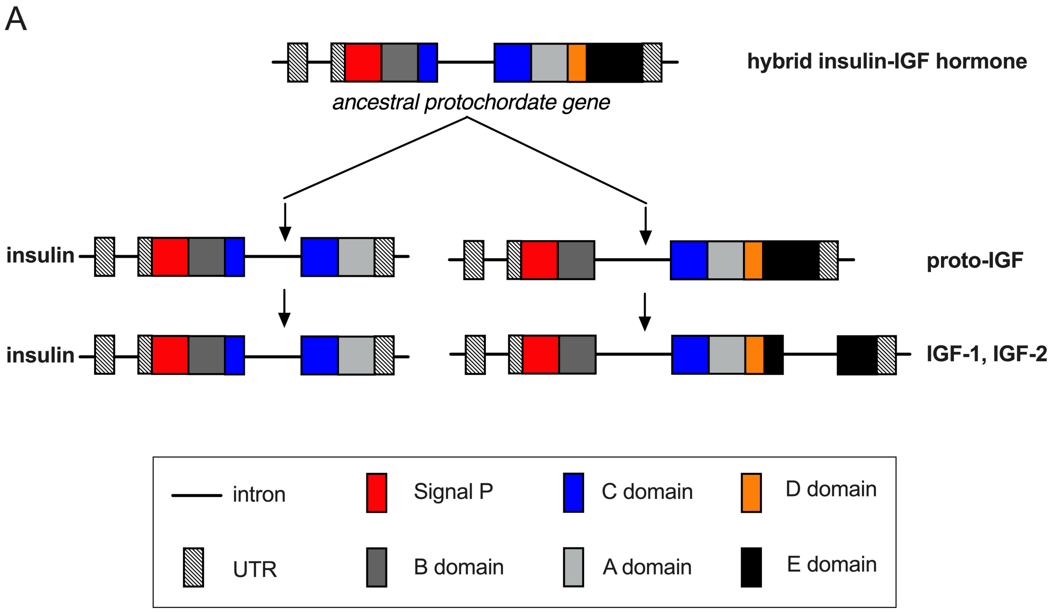

B

B domain

	Insulin 1	10	20		30	IGF-1 1	10	20		30
human	**F**VNQHLCGS	**H**	LVEALYLVCGERGFFYT	P*K*	T	-GPETLCGA	**E**	LVDALQFVCGDRGFYFN	K*P*	T
dog	**F**VNQHLCGS	**H**	LVEALYLVCGERGFFYT	P*K*	A	-GPETLCGA	**E**	LVDALQFVCGDRGFYFN	K*P*	T
cat	**F**VNQHLCGS	**H**	LVEALYLVCGERGFFYT	P*K*	A	-GPETLCGA	**E**	LVDALQFVCGDRGFYFN	K*P*	T
pig	**F**VNQHLCGS	**H**	LVEALYLVCGERGFFYT	P*K*	A	-GPETLCGA	**E**	LVDALQFVCGDRGFYFN	K*P*	T
rat	**F**VKQHLCGP	**H**	LVEALYLVCGERGFFYT	PK	S	-GPETLCGA	**E**	LVDALQFVCGPRGFYFN	K*P*	T
cattle	**F**VNQHLCGS	**H**	LVEALYLVCGERGFFYT	P*K*	A	-GPETLCGA	**E**	LVDALQFVCGDRGFYFN	K*P*	T
sheep	**F**VNQHLCGS	**H**	LVEALYLVCGERGFFYT	P*K*	A	-GPETLCGA	**E**	LVDALQFVCGDRGFYFN	K*P*	T
beluga whale	**F**VNQHLCGS	**H**	LVEALYLVCGERGFFYT	P*K*	A	-GPETLCGA	**E**	LVDALQFVCGDRGFYFN	K*P*	T
chameleon	**L**PNQHLCGS	**H**	LVEALYLVCGDRGFYYS	P*K*	T	-GQETLCGA	**E**	LVDALQFVCGERGFYFS	K*P*	A
common carp	**G**APQHLCGS	**H**	LVDALYLVCGPTGFFYN	P*K*	R	-GPETLCGA	**E**	LVDTLQFVCGDRGFYFS	K*P*	T
chicken	**A**ANQHLCGS	**H**	LVEALYLVCGERGFFYS	P*K*	A	-GPETLCGA	**E**	LVDALQFVCGDRGFYFS	K*P*	T
frog	**L**VNQHLCGS	**H**	LVEALYLVCGDRGFFYY	P*K*	V	-GPETLCGA	**E**	LVDTLQFVCGDRGFYFS	K*P*	T
guinea pig	**F**VSRHLCGS	**N**	LVETLYSVCQDDGFFYI	P*K*	D	-GPETLCGA	**E**	LVDALQFVCGDRGFYFN	K*P*	T
octodon degu	**Y**SSQHLCGS	**N**	LVEALYMTCGRSGFYRP	**H*D***						

Figure S1: Gene structure and evolution of vertebrate insulin and IGFs from an ancestral hybrid gene. (A) Exon-intron organization of the proto-insulin/IGF gene in an ancestral protochordate is hypothesized to contain three exons (boxes) and two introns (bold horizontal lines). Gene duplication and divergence led to vertebrate insulin family (left) and vertebrate IGF family (right). Domain code is shown at bottom. See Chan, S-J et al. (1990, 2000) in main text. **(B)** Alignment of vertebrate B-domain sequences in insulin (left) and IGF-I (right). Residue B1 (bold at far left) is absent in IGF-I (dash). His^{B10} in insulin (red within box) coordinates zinc ions (see Fig. 9) whereas the corresponding Glu9 in IGF-I would repel zinc ions (red in box at right). Divergent hystricomorphic insulins also lack His^{B10}. Dimerization of insulin is stabilized by Pro at position B28 (magenta in box, ordinarily paired with Lys at B29); these residues are inverted in IGF-I (box at far right).

SUPPLEMENTAL TABLE S1

Table S1 – Sites of clinical mutations in proinsulin[a]

amino acid position	*Mutation*	*Type*	*References*
Arg6 [SP6]	Cys, His	MODY (>10y)	(1)
Pro9 [SP9]	Arg	Neonatal diabetes (<1y)	(2)
Leu13 [SP13]	Arg	Neonatal diabetes (<1y)	(3)
Ala24 [SP24]	Asp, Val	Neonatal diabetes (<1y)	(1)
His29 [B5]	Asp, Gln	Neonatal diabetes (<1y)	(1, 4, 5)
	Tyr	MODY (>10y)	
Leu30 [B6]	Pro, Gln, Val, Arg	Neonatal diabetes (<1y)	(1, 5)
	Met	MODY (>10y)	
Cys31 [B7]	Tyr	Neonatal diabetes (<1y)	(6)
Gly32 [B8]	Cys, Ser, Arg, Val	Neonatal diabetes (<1y)	(1, 5, 7)
His34 [B10]	Asp	Hyperproinsulinemia	(1)
Leu35 [B11]	Pro, Gln	Neonatal diabetes (<1y)	(1, 8)
Leu39 [B15]	Pro, Val	Neonatal diabetes (<1y)	(5)
Leu39-Tyr40 delins [B15-B16]	His	Neonatal diabetes (<1y)	(6)
Val42 [B18]	Gly	Neonatal diabetes (<1y)	(9, 10)
	Ala	MODY (>10y)	
Cys43 [B19]	Gly, Ser, Tyr	Neonatal diabetes (<1y)	(1, 5)
	Ala[b]	MODY (>10y)	
Gly44 [B20]	Arg	MODY (>10y)	(11)
Arg46 [B22]	Gln, Stop[a]	MODY (>10y)	(1)
Gly47 [B23]	Val	Neonatal diabetes (<1y)	(1, 5)
	Asp	MODY (>10y)	
Phe48 [B24]	Cys	Neonatal diabetes (<1y)	(1, 12)
	Ser	Hyperinsulinemia	
Phe49 [B25]	Leu	Hyperinsulinemia	(12)
Tyr50 [B26]	Cys	Neonatal diabetes (<1y)	(1)
Pro52 [B28]	Leu	Neonatal diabetes (<1y)	(11)

Arg55 [Cpep-2]	Cys	MODY (>10y)	(1)
Gln78 [Cpep22]	Fs	MODY (>10y)	(13)
Gly84 [Cpep28]	Arg	Neonatal diabetes (<1y)	(1)
Arg89 [Cpep+2]	Cys	Neonatal diabetes (<1y)	(1, 6)
	Pro, Leu, His	Hyperproinsulinemia	
Gly90 [A1]	Cys	Neonatal diabetes (<1y)	(1)
Val92 [A3]	Leu	Hyperinsulinemia	(12)
Glu93 [A4]	Lys	MODY (>10y)	(14)
Cys95 [A6]	Tyr, Ser	Neonatal diabetes (<1y)	(1, 15)
Cys96 [A7]	Arg, Ser, Tyr	Neonatal diabetes (<1y)	(1, 6)
Ser98 [A9]	Cys	Neonatal diabetes (<1y)	(5)
Cys100 [A11]	Tyr	Neonatal diabetes (<1y)	(5)
Ile99-Cys100 ins [B10-B11]	Ser-Ile	Neonatal diabetes (<1y)	(5)
Ser101 [A12]	Cys	Neonatal diabetes (<1y)	(1)
Tyr103 [A14]	Cys	Neonatal diabetes (<1y)	(1)
Gln104 [A15]	Arg	Neonatal diabetes (<1y)	(5)
Leu105 [A16]	Pro	Neonatal diabetes (<1y)	(16)
Asn107 [A18]	Asp	Neonatal diabetes (<1y)	(5)
Tyr108 [A19]	Cys, Asp or Stop	Neonatal diabetes (<1y)	(1, 5)
Cys109 [A20]	Tyr, Phe, Arg	Neonatal diabetes (<1y)	(6)

[a] Residue numbers refer to preproinsulin; positions in the mature A- and B chains are given in brackets. SP and Cpep represents signal peptide and C-peptide respectively.
[b] M. Liu, personal communication.

Supplemental References

1. Støy J, Steiner DF, Park S-Y, Ye H, Philipson LH, Bell GI. Clinical and molecular genetics of neonatal diabetes due to mutations in the insulin gene. Rev Endocr Metab Disord. 2010;11(3):205-15.
2. Laurenzano SE, McFall C, Nguyen L, Savla D, Coufal NG, Wright MS, et al. Neonatal diabetes mellitus due to a novel variant in the INS gene. Mol Case Stud. 2019;5(4):a004085.
3. Hussain S, Mohd Ali J, Jalaludin MY, Harun F. Permanent neonatal diabetes due to a novel insulin signal peptide mutation. Pediatr Diabetes. 2013;14(4):299-303.
4. Donath X, Saint-Martin C, Dubois-Laforgue D, Rajasingham R, Mifsud F, Ciangura C, et al. Next-generation sequencing identifies monogenic diabetes in 16% of patients with late adolescence/adult-onset diabetes selected on a clinical basis: a cross-sectional analysis. BMC Med. 2019;17(1):1-10.
5. Støy J, De Franco E, Ye H, Park S-Y, Bell GI, Hattersley AT. In celebration of a century with insulin–Update of insulin gene mutations in diabetes. Mol Metab. 2021:101280.
6. Liu M, Sun J, Cui J, Chen W, Guo H, Barbetti F, et al. INS-gene mutations: from genetics and beta cell biology to clinical disease. Mol Aspects Med. 2015;42:3-18.
7. Amirruddin NS, Tan YS, Verma CS, Gardner D, Bee YM, Hoon S, et al. 1784-P: Studying the Impact of Heterozygous Human INS Gene Mutation on Pancreatic ß Cell. Am Diabetes Assoc; 2019.
8. Wang T, Ding S, Li S, Guo H, Chen X, Huang Y, et al. A novel mutation in INS gene linked to permanent neonatal diabetes mellitus. Endocrine. 2019;64(3):719-23.
9. Sun F, Du W, Ma J, Gu M, Wang J, Zhu H, et al. A Novel c. 125 T> G (p. Val42Gly) Mutation in The Human INS Gene Leads to Neonatal Diabetes Mellitus via a Decrease in Insulin Synthesis. Exp Clin Endocrinol Diabetes. 2020;128(03):182-9.
10. Piccini B, Artuso R, Lenzi L, Guasti M, Braccesi G, Barni F, et al. Clinical and molecular characterization of a novel INS mutation identified in patients with MODY phenotype. Eur J Med Genet. 2016;59(11):590-5.
11. Wang H, Saint-Martin C, Xu J, Ding L, Wang R, Feng W, et al. Biological behaviors of mutant proinsulin contribute to the phenotypic spectrum of diabetes associated with insulin gene mutations. Mol Cell Endocrinol. 2020;518:111025.
12. Arneth B. Insulin gene mutations and posttranslational and translocation defects: associations with diabetes. Endocrine. 2020;70(3):488-97.
13. Dusatkova L, Dusatkova P, Vosahlo J, Vesela K, Cinek O, Lebl J, et al. Frameshift mutations in the insulin gene leading to prolonged molecule of insulin in two families with Maturity-Onset Diabetes of the Young. Eur J Med Genet. 2015;58(4):230-4.
14. Johnson SR, McGown I, Oppermann U, Conwell LS, Harris M, Duncan EL. A novel INS mutation in a family with maturity-onset diabetes of the young: Variable insulin secretion and putative mechanisms. Pediatr Diabetes. 2018;19(5):905-9.
15. Austin AL, Gatward LFD, Cnop M, Santos G, Andersson D, Sharp S, et al. The KINGS Ins2+/G32S Mouse: A Novel Model of β-Cell Endoplasmic Reticulum Stress and Human Diabetes. Diabetes. 2020;69(12):2667-77.
16. Ortolani F, Piccinno E, Grasso V, Papadia F, Panzeca R, Cortese C, et al. Diabetes associated with dominant insulin gene mutations: outcome of 24-month, sensor-augmented insulin pump treatment. Acta Diabetol. 2016;53(3):499-501.
